# Gas permeability of ice-templated, unidirectional porous ceramics

**DOI:** 10.1080/14686996.2016.1197757

**Published:** 2016-07-18

**Authors:** Jordi Seuba, Sylvain Deville, Christian Guizard, Adam J. Stevenson

**Affiliations:** ^a^ Laboratoire de Synthese et Fonctionnalisation des Ceramiques, UMR3080 CNRS/Saint-Gobain, Cavaillon, France.; ^b^ Institut Europeen des Membranes, Universite de Montpellier 2, Place Eugene Bataillon, Montpellier Cedex 5, France.

**Keywords:** Permeability, porous ceramics, ice-templating, Ergun’s equation, tortuosity, 10 Engineering and Structural materials, 102 Porous / Nanoporous / Nanostructured materials, 107 Glass and ceramic materials, 206 Energy conversion/transport/storage/recovery

## Abstract

We investigate the gas flow behavior of unidirectional porous ceramics processed by ice-templating. The pore volume ranged between 54% and 72% and pore size between 2.9 μm and 19.1 μm. The maximum permeability ($k_{1}=1.39$ $\;\times \;10^{-11}$ m${}^{2}$) was measured in samples with the highest total pore volume (72%) and pore size (19.1 μm). However, we demonstrate that it is possible to achieve a similar permeability ($k_{1}=1.09$ $\;\times \;10^{-11}$ m${}^{2}$) at 54% pore volume by modification of the pore shape. These results were compared with those reported and measured for isotropic porous materials processed by conventional techniques. In unidirectional porous materials tortuosity (τ) is mainly controlled by pore size, unlike in isotropic porous structures where τ is linked to pore volume. Furthermore, we assessed the applicability of Ergun and capillary model in the prediction of permeability and we found that the capillary model accurately describes the gas flow behavior of unidirectional porous materials. Finally, we combined the permeability data obtained here with strength data for these materials to establish links between strength and permeability of ice-templated materials.

## Graphical Abstract

**Figure:**
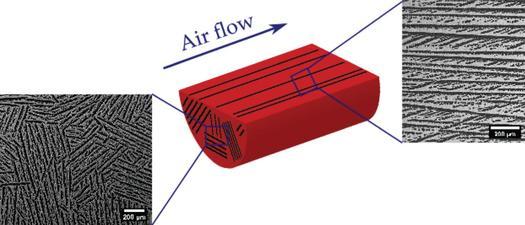

## 1. Introduction

Porous ceramics are used in a variety of applications where high permeability is required, such as filtration of gases and liquids or catalytic supports.[1] The main processing methods are extrusion, ceramic replication of an organic template, and direct foaming of a ceramic suspension. The final properties of porous ceramics are dependent on the processing technique and the resultant microstructure.

In general, ceramics processed by the replica method exhibit accurate porosity control and the pore size is relatively large (200–3000 μm), although the hollow struts characteristic of the replica method have a deleterious effect on strength.[2] In contrast, materials obtained by direct foaming have higher mechanical properties compared to the replica method but larger pore size distribution and consequently a lower permeability.[3] The microstructural effects on permeability of porous ceramics have been extensively studied in different cellular structures. Innocentini et al. [3] compared the effect of pore volume (50–90%) and size (100–900 μm) in materials processed by replica and gel casting. Biasetto et al. [4] conducted a similar study on porous ceramics obtained by organic burn out in a porosity range of 80–90% and pore size 10–150 μm. In both cases, permeability is increased by increasing porosity and pore size compromising the mechanical strength. One possible solution to increase both permeability and strength simultaneously is to engineer a unidirectional porosity (parallel to flow) to minimize the flow resistance without degrading the strength.[5]

The idea of using ice crystals to template porosity in materials and in ceramics in particular have been known for a very long time [6–8], and the underlying principles (the segregation of matter by growing crystals) are more than a century old.[9,10] Ice-templatinghas successfully produced anisotropic porous materials in a simpler manner coupled with a high flexibility to control the pore volume, morphology, and size.[11,12] The process is based on the phase separation that takes place when a colloidal suspension is frozen. After freezing, the solvent is sublimated leaving pores whose morphology is a replica of the ice crystals. Finally, the green body is sintered to consolidate the structure.

In ice-templated materials, Fukasawa et al. [13] studied pressure drop in Si${}_{3}$N${}_{4}$. These structures were specifically designed as filters with fibrous grains protruding into the macropores created by the ice-templating process. The samples ranged in porosity from 62.8% to 69.5% and pore size from 10 to 50 μm. They reported pressure drop data for four samples and found that increasing pore size reduced pressure drop. The data were not compared to known models of permeability, and the effects of the macroporosity was not separated from the effects of the fibrous grains protruding into the pores. Therefore, it is difficult to extend this data and use it to make predictions about permeability in samples with other types of pore morphologies.

Fukushima et al. [14] studied SiC structures with 85% porosity and pore size between 34 and 147 μm. They found that increasing pore size increased permeability. They compared the permeability data to the capillary model.[15] Assuming that tortuosity equaled one, they found that the capillary model matched the data for pore sizes from 34 to 70 μm but overestimated the permeability for samples with an average pore diameter of 147 μm.

Pekor et al. [16] studied the porosity range 43–70% and pore size in the range 6–17 μm in ice-templated alumina. This investigation was focused on the impact of the polyvinyl alcohol (PVA) content on the permeability and found that increasing PVA content increases the non-Darcian component of the flow, most likely because increasing PVA content increases the surface roughness of the ceramic walls. The measured permeabilities were not compared to models.

While there are several studies that discuss permeability in ice-templated ceramics, there is no single study that systematically explores the effects of pore volume, pore size, and pore morphology on permeability. Further, no article connects the mechanical properties of the samples with their permeability, and establishing this link is critical to the industrial application of ice-templated materials.

In this work, we aim to provide a deep understanding of permeability in ice-templated materials across different pore volumes, sizes, and morphologies. We systematically explore the permeability of ice-templated samples with different pore morphologies over the 54–72% porosity range with controlled pore sizes ranging from 2.9 to 19.1 μm. Importantly, we show that the Ergun and the capillary models accurately predict the permeability in materials with unidirectional porosity across the different pore morphologies, porosities, and pore sizes examined here. Finally, we link the permeabilities discussed in this work with mechanical properties obtained in our previous work [17] in order establish links between strength and permeability in unidirectionally porous materials that may help researchers target specific structures to specific application where strength and permeability are critical.

## 2. Experimental procedure

### 2.1. Sample preparation

Porous samples with unidirectional, aligned porosity were prepared by ice-templating. Suspensions were prepared by mixing distilled water with 0.75 wt% of dispersant (Prox B03, Synthron, Levallois-Paris, France), 3 wt% of PVA (PVA2810, Wacker, Burghausen, Germany) as a binder, and 3 mol% yttria-stabilized zirconia (TZ-3YS, Tosoh, Tokyo, Japan) at different solids loading ranging from 50 wt% (14.7 vol.%) to 65 wt% (24.2 vol.%). In some suspensions, zirconium acetate (20 g l${}^{-1}$) was added to the slurry to modify the pore morphology.[18] All suspensions were ball-milled with alumina-milling media for a minimum of 18 h to achieve a good dispersion.

Afterwards, 10 ml of slurry was poured into a PTFE mold (20 mm diameter and 25 mm height) placed on top of a copper plate. The top of the mold was exposed to air and kept at room temperature. Samples were unidirectionally frozen from the bottom to the top circulating silicone oil by the freezing plate. Temperature of the oil was regulated by a cryothermostat (Model CC 905, Hubert, Offenburg, Germany). The cooling rate was set at 2 ${}^{\circ }$C min${}^{-1}$. A faster cooling rate was achieved by dipping a copper rod with the mold on top in a liquid nitrogen bath. An isolating panel was placed in between the mold and the top of the bath to avoid the direct contact of the vaporized liquid nitrogen with the mold. The cooling rate was monitored with a thermocouple and it was determined to be 25 ${}^{\circ }$C min${}^{-1}$ on average. After solidification, samples were removed from their molds and sublimated for at least 48 h in a commercial freeze-dryer (Free Zone 2.5 Plus, Labconco, Kansas City, MO, USA) until the solvent was completely removed. The sintering cycle involved removal of organic components, which was achieved by heating the samples from room temperature to 500 ${}^{\circ }$C at a rate of 3 ${}^{\circ }$C min${}^{-1}$ and holding them at 500 ${}^{\circ }$C for 5 h. Samples were sintered at 1400 ${}^{\circ }$C for 3 h using a heating and cooling rate of 5 ${}^{\circ }$C min${}^{-1}$. Finally, we cut 2 mm off the bottom of each sample to remove the area with randomly oriented porosity [19] and further minimize the pore size gradient between the bottom and the top, thereby testing the part with continuous porosity and with an almost constant cross section. The top of the specimens was also removed to avoid possible artifacts that may appear during the demolding.

To evaluate the effect of pore directionality, samples with randomly oriented porosity were prepared using pore formers. The same zirconia powder was mixed with commercially available pore former polypropylene with a mean particle size ($d_{50}$) of 45–55 μm and a maximum particle size ($d_{100}$) of 105 μm (Propyltex 140S, Micro Powders Inc., Tarrytown, NY, USA) at 50 wt% and mixed with distilled water. The slurry was magnetically stirred and ball milled for a minimum of 24 h to break up the agglomerates. Afterwards, the slurry was frozen by dipping the container in liquid nitrogen and freeze-dried to obtain an homogeneous mix. Then, 8 g of the obtained powder was pressed at 8 MPa in a mold of 20 mm diameter. The sintering temperature and dwell time were the same as those used for the ice-templated samples. To ensure a proper burn-out and crack free samples an extra hold of 1 h was added at 900 ${}^{\circ }$C.

### 2.2. Sample characterization

The overall porosity P(%) was calculated based on the mass (*m*) and volume (*V*) of the samples with respect to that of fully dense TZ-3YS ($\rho_{ysz}=5.8gcm^{-3}$), as:

(1) $$\begin{array}{cc} \rho_{rel} & =\frac{\rho }{\rho_{ysz}}=\frac{mV^{-1}}{\rho_{ysz}} \end{array}$$

(2) $$\begin{array}{cc} P(\%) & =(1-\rho_{rel})\times 100\% \end{array}$$

Pore size was evaluated as the width of the lamellae and quantified using two techniques: image analysis and mercury intrusion porosimetry. Image analysis was performed in at least five cross-sections perpendicular to the freezing direction (height was kept constant at 7 mm from the bottom of the sample). Some samples were sectioned parallel to the freezing direction to evaluate the directionality of the pores. All the images were obtained using a scanning electron microscope (Nova NanoSEM 230, FEI, Hillsboro, OR, USA). Furthermore, pore size results were confirmed by mercury intrusion porosimetry (AutoPore IV 9500, Micromeritics, Norcross, GA, USA) with an applied pressure up to 0.31 bar.

**Figure 1.**
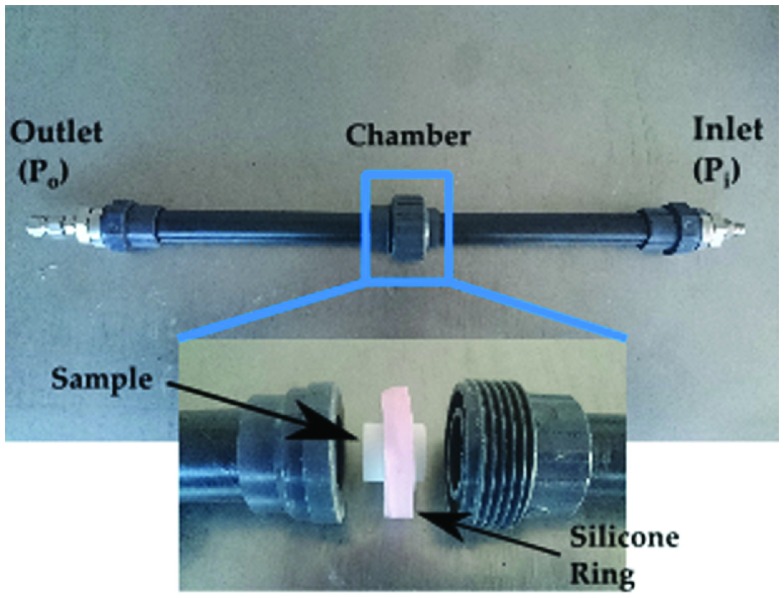
Setup used to measure pressure drop.

Gas permeability was measured using a custom built equipment and is shown in Figure 1. Synthetic air was passed through the sample which was held by a silicone ring. Two sensors were placed before and after to record the inlet ($P_{i}$) and outlet pressure ($P_{o}$) at room temperature. The tested samples had a thickness (*L*) of 12–15 mm and a diameter 15 mm. In the case of ice-templated specimens, pores were always aligned parallel to the flow during the test.

Permeability was evaluated using the Forchheimer’s equation adapted for compressible fluids, Equation (3):

(3) $$\begin{array}{c} \frac{\Delta P}{L}=\frac{{P_{i}}^{2}-{P_{o}}^{2}}{2P_{o}L}=\frac{\mu }{k_{1}}\nu_{s}+\frac{\rho }{k_{2}}{\nu_{s}}^{2} \end{array}$$

where L is the thickness of the sample (parallel to the flow), μ and ρ are the viscosity and the density of the fluid (air at 25 ${}^{\circ }$C), ΔP is the pressure drop, $k_{1}$ and $k_{2}$ are the Darcian and non-Darcian permeabilities, and $\nu_{s}$ is the fluid velocity (air flow divided by the cross-sectional area of the sample). The maximum air flow ($\nu_{s}$) generated was 2 m s${}^{-1}$ and ΔP did not exceed 350 MPa.

## 3. Results and discussion

### 3.1. Microstructure of ice-templated samples

**Figure 2.**
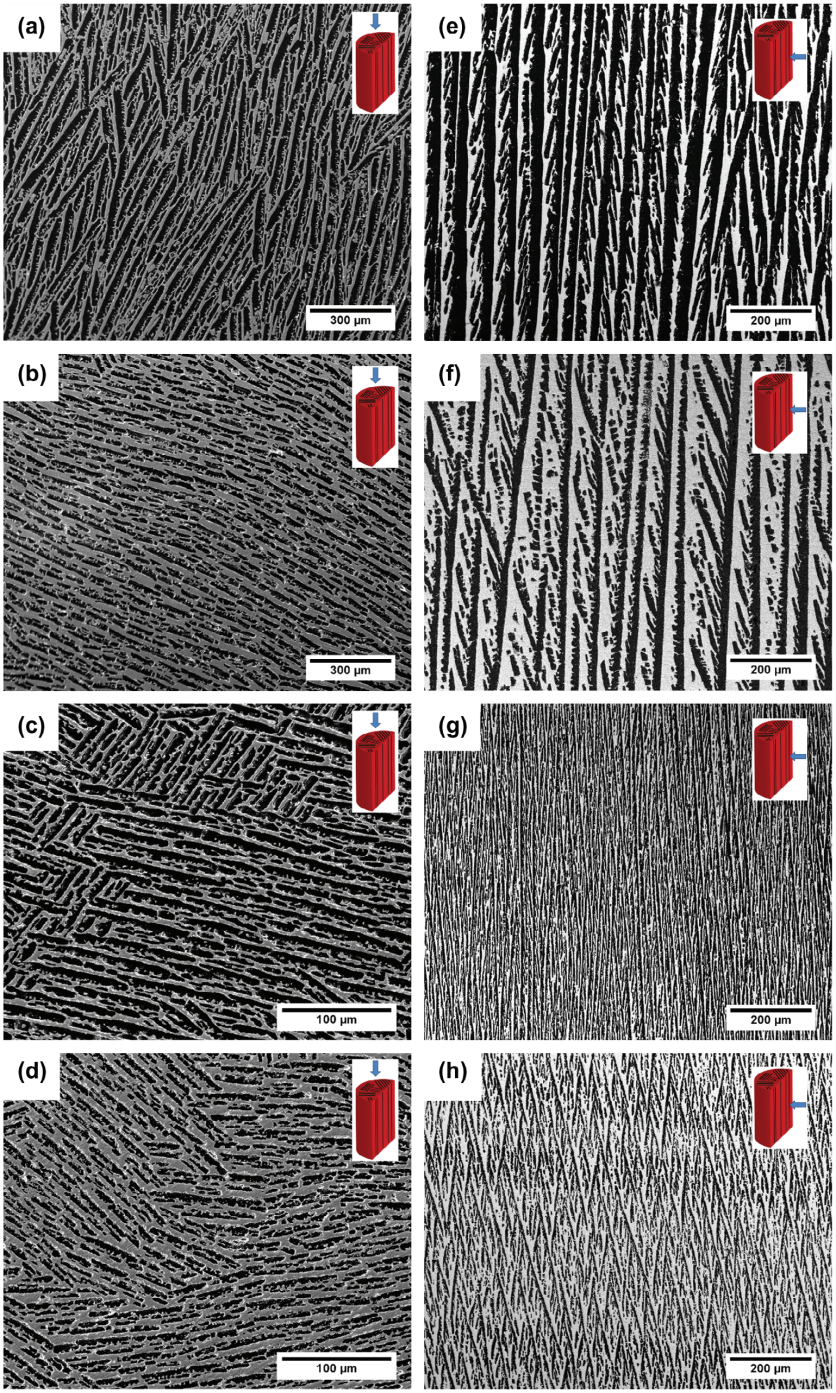
Microstructures of ice-templated samples listed in Table 1, with cross-sections perpendicular to the freezing direction: (a) S1, (b) S4, (c) S5, and (d) S8; and parallel to it: (e) S1, (f) S4, (g) S5, and (h) S8.

Figure 2(a)–(d) shows cross sections perpendicular to the solidification front for the samples S1, S4, S5, and S8 in Table 1. All of them exhibit a lamellar pore morphology characteristic of ice-templated samples with water used as a solvent and without ice-shaping additives incorporated to the initial slurry. Total pore volume and pore size were almost independently controlled by the initial solids loading and cooling rate respectively. Figures 2(a) and (b) show the microstructures obtained in two samples frozen at identical cooling rate (2 ${}^{\circ }$C min${}^{-1}$) but different solids loading (50 and 65 wt%). The modification of the solids loading causes a variation on the relative density of the samples, and the total pore volume changes accordingly. Increasing the solids loading from 50 wt% (14.7 vol.%) to 65 wt% (24.2 vol.%) causes a decrease in total pore volume from 72% to 54%. The same effect has also been observed at higher freezing rate (Figure 2(c) and (d), but the pore size obtained was smaller. For example, when freezing temperature increased from 2 to 25 ${}^{\circ }$C min${}^{-1}$, mean pore size ($d_{c}$) decreased from 20 ± 9 μm (S1) to 5 ± 2 μm (S5); however, pore volume remained unchanged, at 72%.

Table 1 also highlights the effect of solids loading on pore size. Specimens ice-templated with 50 wt% solids loading exhibit a pore size remarkably larger (20 ± 9 μm) than those ice-templated with 65 wt% (14 ± 5 μm). This effect is caused by the increasing number of ceramics particles at higher solids loading that constrain the crystal growth, and consequently decrease the mean pore size. Although the pore size reduction caused by the solids loading holds a second order of importance compared with the cooling rate, it is important to consider it because certainly might affect the permeability.

**Table 1.** Properties of ice-templated samples. ${d_{c}}_{Hg}$ and ${d_{c}}_{IA}$ refer to the mean pore size measured by mercury porosimetry and image analysis respectively. $R^{2}$ corresponds to the correlation coefficient of the trend lines shown in Figure 4.

Sample	Solids loading (wt%)	Solids loading (vol.%)	Cooling rate (${}^{\circ }$C min${}^{-1}$)	Porosity (%)	Mean ${d_{c}}_{Hg}$ (μm)	Mean ${d_{c}}_{IA}$ (μm)	$k_{1}$ (m${}^{2}$)	$R^{2}$
S1	50%	14.7%	2	72%	19.1	20 ± 9	1.39$\;\times \;10^{-11}$	0.999
S2	55%	17.4%	2	67%	17.5	18 ± 7	9.44$\;\times \;10^{-12}$	0.999
S3	60%	20.6%	2	60%	14.2	14 ± 6	5.95$\;\times \;10^{-12}$	0.999
S4	65%	24.2%	2	54%	8.3	14 ± 5	2.17$\;\times \;10^{-12}$	0.998
S5	50%	14.7%	25	72%	5.4	5 ± 2	1.08$\;\times \;10^{-13}$	0.999
S6	55%	17.4%	25	68%	4.3	4 ± 2	7.99$\;\times \;10^{-14}$	0.999
S7	60%	20.6%	25	60%	3.9	4 ± 2	4.27$\;\times \;10^{-14}$	0.999
S8	65%	24.2%	25	55%	2.9	3 ± 1	2.36$\;\times \;10^{-14}$	0.999

Figure 2(e)–(h) show the microstructures of the same ice-templated samples parallel to the solidification front. In all cases, pores are continuous with an almost constant cross-section, independently of solids loading (Figure 2(e) and (f)) and cooling rate (Figure 2(g) and (h). Moreover, all the samples exhibited the characteristic dendritic growth of ice-templated materials.

Since air flow in porous materials is a volumetric phenomenon and is highly sensitive to pore size, a complementary evaluation to image analysis was performed by mercury porosimetry. This technique probes the entire volume of the sample in contrast to the local measurements obtained by image analysis.

**Figure 3.**
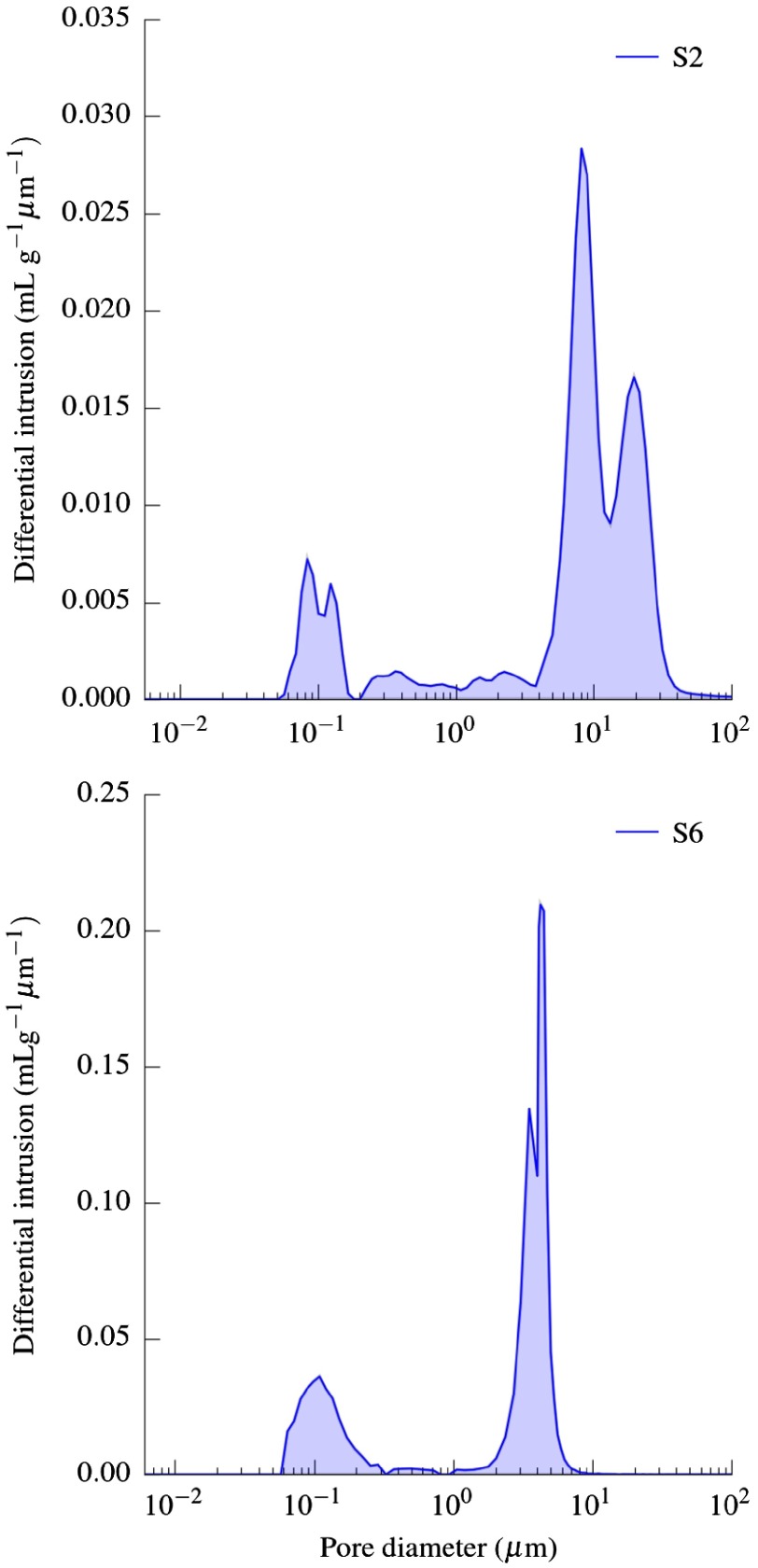
Pore size distribution obtained by mercury intrusion porosimetry. S2 and S6 refer to specimens described in Table 1. In both cases P(%) = 68%.

Figure 3 shows the pore size distribution of two representative samples (S2 and S6) described in Table 1. As extensively reported [20–24], porous ceramics processed by ice-templating usually exhibited a bimodal pore distribution. In both samples, larger pore size corresponds to the open unidirectional porosity created by the sublimation of the ice crystals and, thus mainly controlled by the solids loading. The double peak observed at around 20 μm for S2 and 4 μm for S6 can be related with the primary and secondary arms characteristic of the ice crystal during the dendritic growth. Alternatively, the smaller peak (around 0.1 μm) corresponds to porosity located in the ceramic walls and can be tailored by the sintering temperature and powder characteristics.

**Figure 4.**
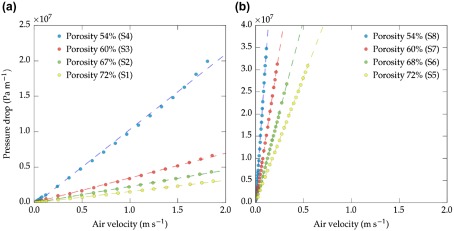
Effect of air velocity ($\nu_{s}$) on pressure drop (ΔP) for different pore volumes parallel to the flow. Samples were ice-templated samples at (a) 2 ${}^{\circ }$C min${}^{-1}$ and (b) 25 ${}^{\circ }$C min${}^{-1}$.

Results obtained by both techniques, image analysis and mercury intrusion porosimetry, are in excellent agreement, as shown Table 1. For example, S1 exhibits a pore size of 19.1 μm obtained by mercury porosimetry, and remarkably close to 20 ± 9 obtained by image analysis. In general, the pore size measured by mercury intrusion should show smaller size than those measured by image analysis, due to the effect of bottle-necks. However, in these ice-templated unidirectional porous ceramics, the cross-section of the pores is constant, so the presence of bottle-necks tends to be minimized. The only exception is the specimen S4 that gives a slightly lower pore size value, probably caused by the presence of volumetric defects originated by the high solids loading.

### 3.2. Pressure drop and permeability

Figure 4(a) and (b) show the variation of pressure drop (ΔP) with air velocity ($\nu_{s}$) for the specimens S1–S8 described in Table 1. Obviously, increasing $\nu_{s}$ leads to increase ΔP. However, the different slopes exhibitedby all groups highlight the importance of pore volume and pore size on the gas flow resistance parallel to pore orientation and hence pressure drop.

All samples followed a linear relationship between ΔP and $\nu_{s}$. This behavior can be explained based on the Forchheimer’s equation (Equation (3)). The first term ($\mu \nu_{s}/k_{1}$) in Equation (3) considers the viscous energy losses caused by the friction between fluid layers, and the second term ($\rho \nu_{s}^{2}/k_{2}$) accounts for the inertial effects created by turbulence and variations in the direction and acceleration of the fluid.[25] The excellent linear correlation coefficient ($R^{2}$ > 0.998 in Table 1) supports the hypothesis that all the tests were performed in viscous flow regime and thus, the determination of permeability is only subjected to the reduced form of Equation (3), i.e. Darcy’s law:

(4) $$\begin{array}{c} \frac{\Delta P}{L}=\frac{{P_{i}}^{2}-{P_{o}}^{2}}{2P_{o}L}=\frac{\mu }{k_{1}}\nu_{s} \end{array}$$

Nevertheless, careful considerations should be made if $k_{1}$ is intended to be applied in systems with higher air flow. In these conditions the inertial effects and turbulence become important and both constants ($k_{1}$ and $k_{2}$) are required to describe the behavior.[26]

**Figure 5.**
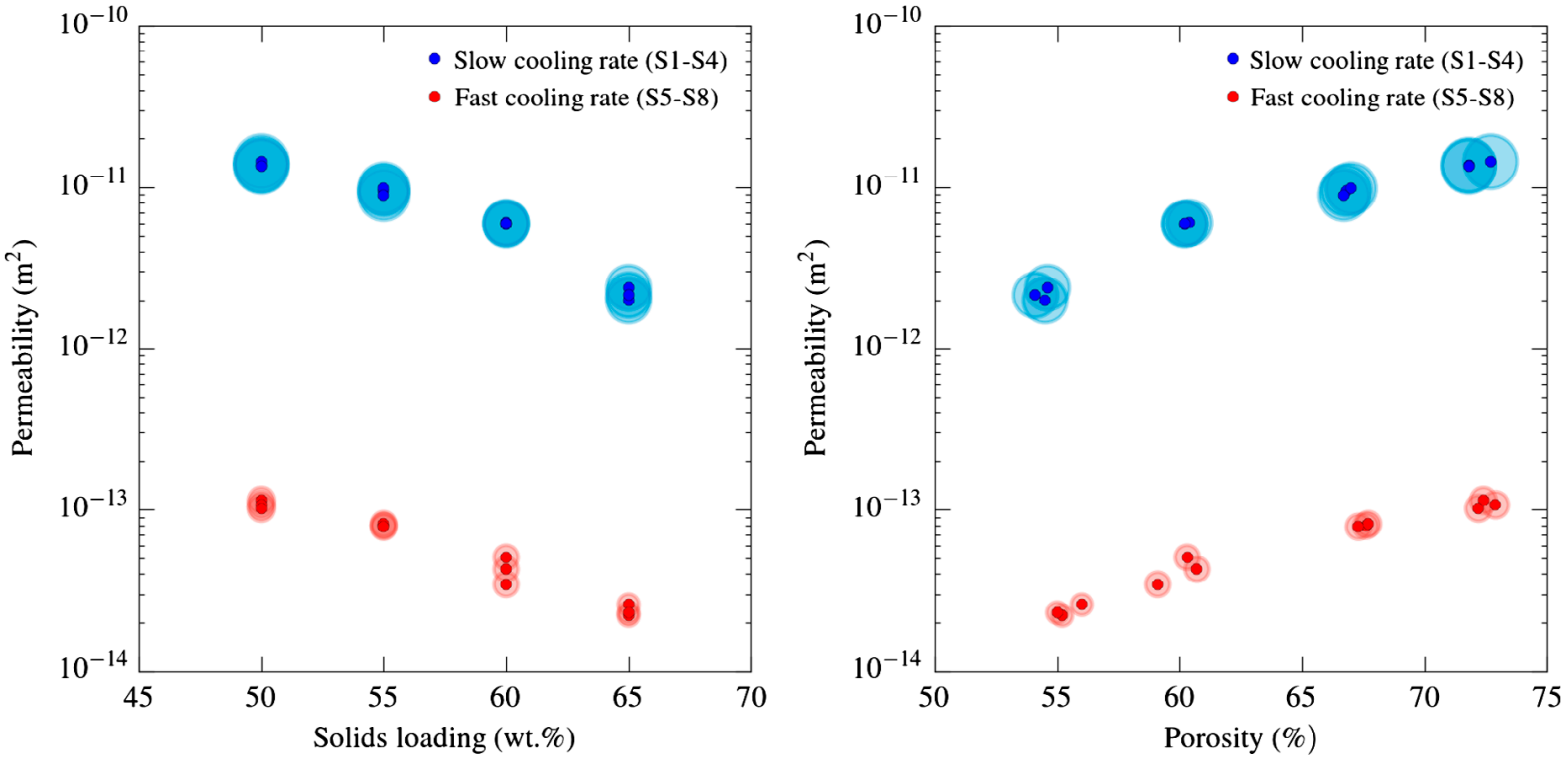
Effect of solids loading and freezing rate on permeability. Experimental and morphological parameters are specified in Table 1. Slow and fast cooling rate correspond to 2 and 25 ${}^{\circ }$C min${}^{-1}$ respectively. Circles around the markers are proportional to pore size.

Figure 5 and Table 1 show the effect of initial solids loading (and hence porosity) and cooling rate on permeability. Decreasing the solids loading causes an increase in $k_{1}$ that can be correlated with an increase in total pore volume as well as a slight increase in pore size. The specimens frozen faster are remarkably less permeable. The main explanation for this behavior relies on the important reduction in pore size observed at these freezing conditions. The fact that the decrease in permeability is around two orders of magnitude in samples with the same total pore volume emphasizes the great importance of pore size to maximize the fluid flow.

The values obtained in this work are in agreement with those found in the literature for ice-templated samples with similar microstructure. Pekor et al. [16] reported a permeability of 1.0$\;\times \;10^{-11}$ m${}^{2}$ for ice-templated alumina with 70% porosity and pore size around 17 μm. Fukushima et al. [14] obtained a slightly higher $k_{1}$ (2.27$\;\times \;10^{-11}$ m${}^{2}$) for SiC prepared by gelation freezing method but with a remarkably higher pore volume and pore size (86% and 34 μm).

**Figure 6.**
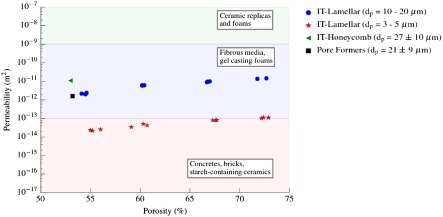
Comparison of the permeabilities that can be achieved by the most common processing techniques [1]. Description of specimens labeled as ‘IT-Lamellar’ is shown in Table 1. Blue and red markers correspond to samples S1–S4 and S5–S8 respectively.

Comparing the permeability exhibited by ice-templated materials with other macroporous materials obtained by different techniques, we can see in Figure 6 that the former falls in the wide range exhibited by gelcasted foams and much lower than those typically exhibited by reticulated foams. However, the total pore volume, and more importantly the pore size of this type of cellular materials, is much higher compared with those obtained by ice-templating. For example, Innocentini et al. [3] reported a $k_{1}$ of 1.5$\;\times \;10^{-10}$ m${}^{2}$ for a a porous alumina processed by replica method with 70% porosity and 200 μm pore size and a permeability of 5$\;\times \;10^{-11}$ m${}^{2}$ for a porous alumina prepared by gelcasting with the same pore volume and 125 μm pore size.

**Figure 7.**
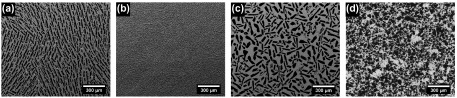
Representative microstructures of specimens shown in Figure 6. (a) Ice-templated, lamellar morphology, and slow cooling rate; (b) ice-templated, lamellar morphology, and fast cooling rate; (c) ice-templated, honeycomb morphology, and slow cooling rate; (d) pore formers. All of them exhibited a similar pore volume (P ≈ 54%).

**Figure 8.**
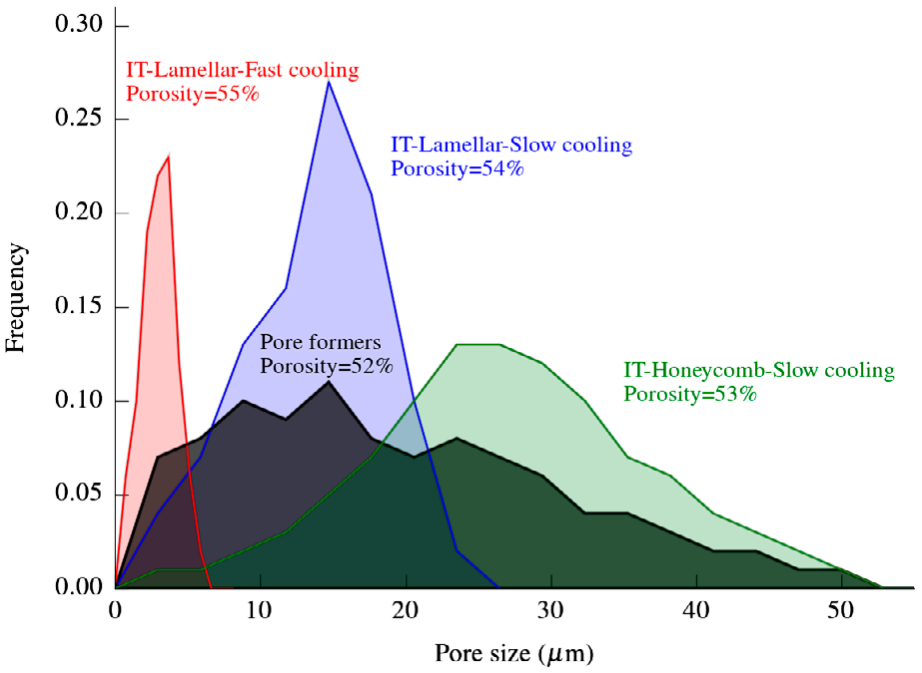
Pore size distributions obtained by image analysis of different ice-templated structures compared with sacrificial method (pore formers). Microstructures are shown in Figure 7.

Additionally, we evaluated the effect of pore directionality (i.e. ‘Pore Formers’ in Figure 6) and morphology (i.e. ‘IT-Honeycomb’ in Figure 6) on permeability. The first group consists of samples with isotropic porosity processed by burn out of organic pore formers (Figure 7(d)), and the second group comprises ice-templated samples with zirconium acetate (ZRA) added to the original slurry. The addition of ZRA turns the pore morphology into a honeycomb-like structure with smooth surfaces (Figure 7(c)). As Figure 8 shows, this porous structure posses a larger mean pore size and a broader distribution than the typical lamellar ice-templated morphology (Figure 7(a)). This variation in pore size distribution causes an increase in permeability of almost one order of magnitude at comparable total pore volume (Figure 6). In contrast, the specimens processed by pore formers have a similar permeability as the ice-templated specimens with lamellar morphology and therefore an apparent no effect of pore tortuosity (Figure 6). However, although the mean pore size is similar in both samples, the distribution tends to be more spread in materials processed by pore formers (Figure 8). The presence of these big pore clusters might facilitate the air flow through the samples and consequently mask the effect of pore directionality. Although the presence of larger isolated pores certainly increases the permeability, they can become stress concentrators and therefore affect the mechanical integrity of the system.

Most of the important industrial applications involving porous materials are subjected to a trade-off between permeability and strength [27]. Since both properties are inversely related to total pore volume, other strategies different than increasing/decreasing this parameter are necessary to improve both properties simultaneously.

**Figure 9.**
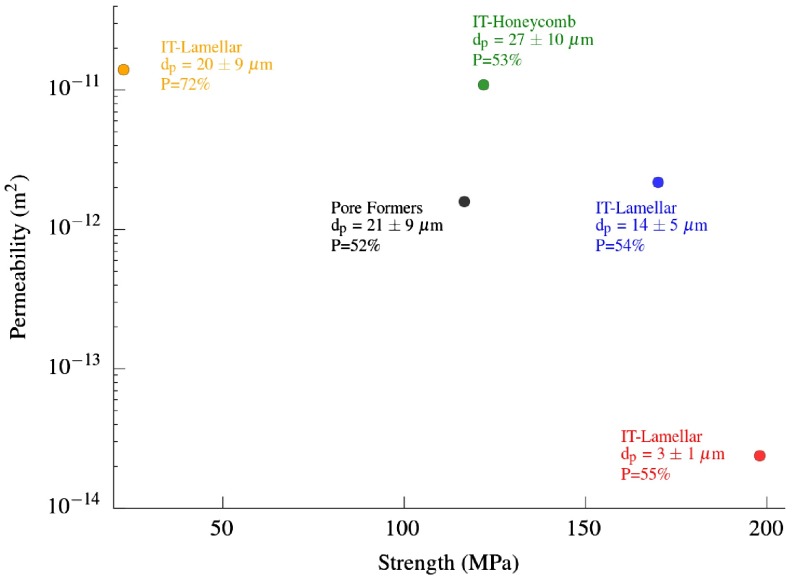
Compressive strength and permeability of ice-templated (IT) and pore formers samples processed in this work. $d_{p}$ and P refer to pore size and porosity respectively. Pore size distributions are shown in Figure 8.

Figure 9 shows the effect of microstructural variations in permeability studied in this work along with their corresponding compressive strength reported in a previous article.[17] As expected, the ice-templated specimens with smaller pore size have the highest strength. This behavior might be caused by the larger connectivity among the walls that prevents the structure from buckling and shearing stresses.[24] Unfortunately, this pore structure also exhibits the lowest permeability. On the other hand, ice-templated samples frozen at slow cooling rate, either lamellar or honeycomb morphology, exhibited both a higher compressive strength and permeability than the samples prepared by pore formers. Therefore, the unidirectionally aligned porosity of ice-templated materials seems to be more optimized to maximize mechanical stability and permeability than the randomly oriented porosity exhibited by materials processed by pore formers.

The ice-templated specimens with honeycomb morphology reached permeability values comparable to ice-templated samples with P = 72% coupled with a compressive strength around one order of magnitude higher. This microstructure exhibits higher permeability than required for diesel particulate filters,[28] and it could be of particular interest in applications that require a reasonably high mass flow and low pressure drop (e.g. membranes for water/air filtration and catalytic supports).[29]

### 3.3. Prediction by models

Most of the models used to predict the permeability constant ($k_{1}$) are extracted from correlations originally developed for granular beds and are dependent on the volumetric void fraction (ϵ), and the equivalent particle size ($d_{p}$). The most common expression was developed by Ergun [30] (Equation (5)) and it has been extensively applied to all types of porous media.[31–33]

(5) $$\begin{array}{c} k_{1}=\frac{\epsilon^{3}{d_{p}}^{2}}{150{\left(1-\epsilon \right)}^{2}} \end{array}$$

**Figure 10.**
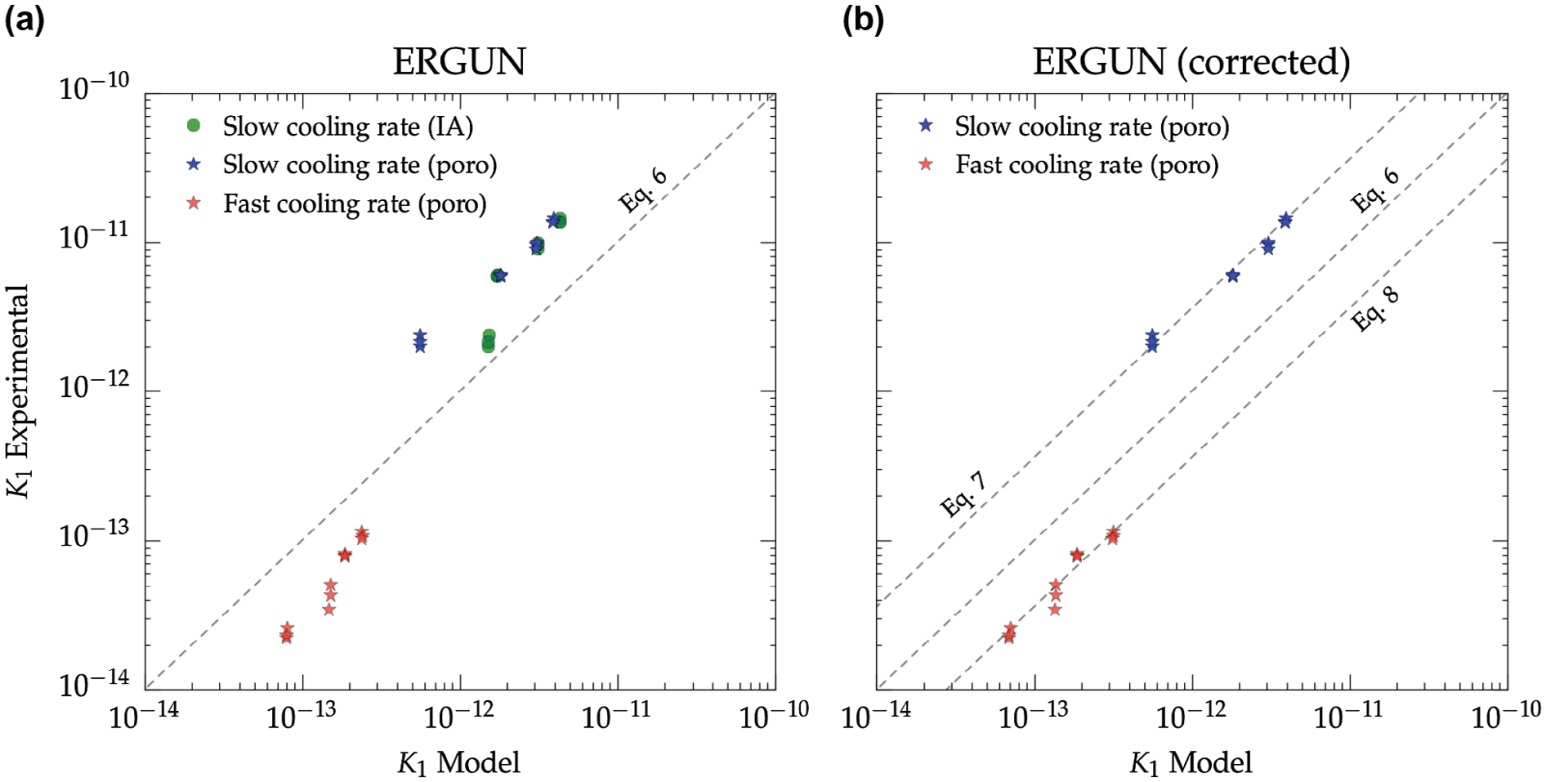
Comparison between the experimental permeability data and the values predicted by the original (A) and modified (B) Ergun equations. Two different methods were used to evaluated the pore size, image analysis (IA) and mercury porosimetry (poro). Samples labeled as ‘Slow cooling rate’ and ‘Fast cooling rate’ correspond to S1–S4 and S5–S8 in Table 1 respectively.

However, this approach is hard to apply to cellular materials due to the difficulties in extracting the characteristic parameter that could replace the particle size ($d_{p}$). One solution consists on replacing $d_{p}$ by an equivalent pore size ($d_{c}$) [34] and then, $k_{1}$ can be rewritten as:

(6) $$\begin{array}{c} k_{1}=\frac{2.25}{150}\epsilon {d_{c}}^{2} \end{array}$$

The experimental values of $k_{1}$ for ice-templated samples frozen at 2 ${}^{\circ }$C min${}^{-1}$ and 25 ${}^{\circ }$C min${}^{-1}$ were compared with those predicted by Equation (6). Since the evaluation of permeability by Ergun’s equations is highly sensitive to the equivalent pore size ($d_{c}$), the assessment of the model was conducted with the pore sizes measured by image analysis and mercury porosimetry. As Figure 10(a) shows, all the experimental points diverge from the prediction of the Ergun model (dashed line) independently of the technique used to characterize the pore size. The only exceptions are the specimens frozen at slow cooling rate and lower permeability which seemingly exhibited a better fitting when the pore size was obtained by image analysis. However, this result clearly deviates from the tendency displayed by the other samples and can be attributed to the increasing presence of closed pores found at high solids loading (S4 in Table 1).

The main factor that explains the deviation between experimental and calculated values of $k_{1}$ relies on the semi-empirical nature of Ergun expression. The value of 150 in Equation (6) was originally determined on different experiments on highly tortuous media, and thus it might not be valid for radically different porous structures like unidirectional porous materials. Nevertheless, since it is an expression broadly used and for the sake of comparison, we corrected the original Ergun equation (Equation (6)) to represent the experimental data obtained in this work (Figure 10(B)). Equation (7) describes the permeability of samples frozen at 2 ${}^{\circ }$C min${}^{-1}$ and Equation (8) for samples frozen at 25 ${}^{\circ }$C min${}^{-1}$.

(7) $$\begin{array}{cc} k_{1} & =\frac{2.25}{42}\epsilon {d_{c}}^{2} \end{array}$$

(8) $$\begin{array}{cc} k_{1} & =\frac{2.25}{407}\epsilon {d_{c}}^{2} \end{array}$$

The microstructure of ice-templated materials better resemble a capillary system where pores are aligned parallel to the gas flow than a porous granular media. The maximum permeability in an ideal capillary model is described by Equation (9):[15]

(9) $$\begin{array}{c} K_{capillary}=\frac{\phi d_{c}^{2}}{\tau 32} \end{array}$$

where ϕ, $d_{c}$, and τ are porosity, pore size, and tortuosity respectively.

**Figure 11.**
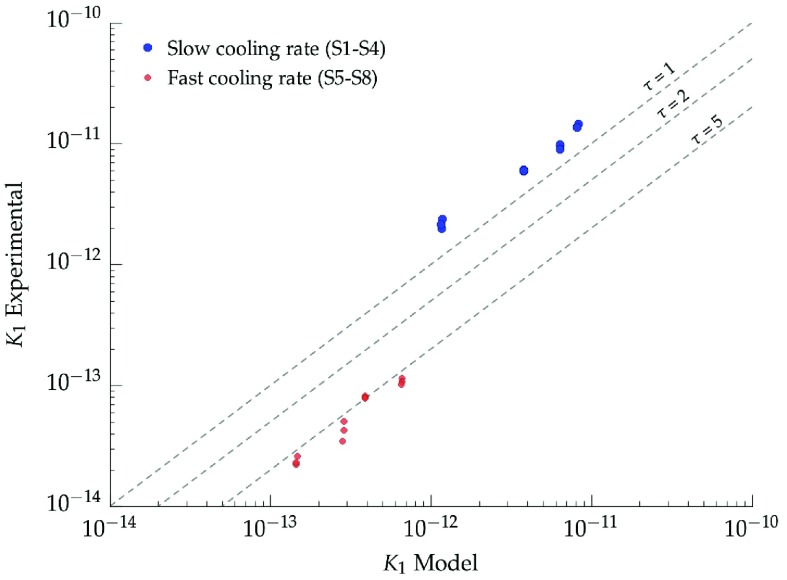
Comparison between the experimental permeability data and the values predicted by the capillary model.

The capillary permeability ($K_{capillary}$) of ice-templated samples was calculated based on Equation (9) and using the measurements of porosity and pore size obtained by mercury porosimetry in Table 1. Figure 11 shows the experimental values of $k_{1}$ compared with those predicted by the model. The permeability of ice-templated samples frozen at slow cooling rate (2 ${}^{\circ }$C min${}^{-1}$) is close to the maximum obtained when τ=1. This result indicates that the porosity of these samples is almost continuous and the capillary model is thus a good descriptor of permeability in these samples. However, the permeability of ice-templated materials is not always predicted by the ideal case of Equation (9) where τ=1. For example, the best fit for high freezing rate specimens (25 ${}^{\circ }$C min${}^{-1}$) is exhibited around τ=5. Usually, in isotropic porous materials, tortuosity tends to decrease when pore volume increases.[35] Unlike in isotropic samples, tortuosity of ice-templated materials remains constant in a wide porosity range (50–70%) but is affected by pore size.

## 4. Conclusions

The air permeability of ice-templated materials was evaluated as a function of different morphological parameters such as pore volume, size, and morphology. The results showed that pore size is the main parameter controlling the pressure drop and is therefore of capital importance to increase the permeability of porous materials without a detrimental effect on the mechanical stability. Based on this knowledge we produced ice-templated samples with a 53% pore volume and a permeability equivalent to 72% pore volume through a pore shape modification. This reduction on total pore volume allowed samples to be obtained with a high resistance coupled with a high permeability, two properties seemingly inversely related.

Microstructure control using ice-templating allows us to tailor the mechanical and gas flow properties almost independently and consequently could be of potential application in products such as filters or catalytic supports. Using a pressure drop test we evaluated the tortuosity of ice-templated samples and we obtained a value remarkably close to the ideal case (τ=1). In this case, tortuosity remained constant for a pore volume increase and it was mainly controlled by pore size, unlike in isotropic porous structures. Finally, we also demonstrate that the permeability of unidirectional porous materials can be described by the capillary model in a wide porosity range (50–70%). However, this study only covers the laminar regime and a deeper understanding of the microstructural effects on a turbulent flow is still required in these types of materials.
